# Exploring Wavefront Detection in Imaging Systems with Rectangular Apertures Using Phase Diversity

**DOI:** 10.3390/s24041191

**Published:** 2024-02-11

**Authors:** Yibo Li, Jiang Guo, Rengcong Liu

**Affiliations:** 1 Changchun Institute of Optics, Fine Mechanics and Physics, Chinese Academy of Sciences, Changchun 130033, China; liyibo221@mails.ucas.ac.cn (Y.L.); liurengcong22@mails.ucas.ac.cn (R.L.); 2 University of Chinese Academy of Sciences, Beijing 100049, China

**Keywords:** Legendre polynomial, phase diversity method, rectangular aperture imaging system, space telescope, wavefront detection

## Abstract

The attainment of a substantial aperture in the rotating synthetic aperture imaging system involves the rotation of a slender rectangular primary mirror. This constitutes a pivotal avenue of exploration in space telescope research. Due to the considerable aspect ratio of the primary mirror, environmental disturbances can significantly impact its surface shape. Active optical technology can rectify surface shape irregularities through the detection of wavefront information. The Phase Diversity (PD) method utilizes images captured by the imaging system to compute wavefront information. In this study, the PD method is applied to rotating synthetic and other rectangular aperture imaging systems, employing Legendre polynomials to model the wavefront. The study delved into the ramifications stemming from the aperture aspect ratio and aberration size.

## 1. Introduction

The size of the aperture plays a crucial role in determining the performance of space telescopes. A larger-aperture system can capture high-resolution images of distant and faint celestial objects. However, due to constraints in carrying capacity, the diameter of a space telescope with a conventional monolithic primary is generally limited to 4 m. To address this limitation, there have been developments in creating primary mirrors using new materials and technologies that simulate large apertures, such as the membrane mirror [1,2], segmented mirror [3,4], and rotating synthetic aperture (RSA) imaging systems [5].

The membrane mirror has a very low surface density and can be easily folded and stored. There are two main types of membrane mirror: inflatable [6] and electrostatic [7]. Inflatable membrane mirrors have been used in radar bands, but due to the fluidity of the gas, the surface shape is difficult to control and cannot be applied to optical bands. Electrostatic thin film mirrors can achieve precise shape control, but their support and control structures are so complex that it will take a long time before they are applied in orbit. The segmented mirror is more advanced. It combines several smaller sub-mirrors into an equivalent main mirror. However, this technology needs accurate adjustment of the sub-mirror attitude, and the equivalent main mirror’s surface shape accuracy is inferior to the traditional monolithic mirror. It is only suitable for the infrared band at present.

The RSA Telescope, backed by the U.S. government, Northrop Grumman, and Raytheon, is being developed with the goal of achieving a 20-m equivalent diameter. Featuring a narrow rectangular primary mirror, this monolithic mirror can be positioned vertically at launch, maximizing the height of the launch section and surpassing the carrying limitations. Throughout the imaging process, the primary mirror undergoes rotation around the center. Through the fusion of images captured at various angles, a high-resolution image is generated, utilizing the long side of the rectangular primary mirror as the equivalent diameter. Harbin Institute of Technology carried out a comprehensive study on this technology using simulation and experiments [8,9,10], explained the imaging principle of the RSA system, and analyzed the impact of factors such as the pupil aspect ratio and the rotation of the primary mirror. Yagnyatinskiy [11] simulated the influence function of the actuator in the rectangular deformable mirror and investigated the correction technology of the rectangular mirror surface. However, no relevant research on the wavefront detection technology of the rectangular aperture imaging system has been reported.

For the rotating pupil imaging system, the main sources of error during the imaging process include the wavefront aberration induced by the primary mirror deformation, the detector noise, and motion blur on the PSF caused by the rotation of the primary mirror. After entering orbit, the large surface error of the primary mirror requires preliminary detection and correction before rotating. During this process, the error caused by the primary mirror rotation can be ignored.

The Marechal criterion states that an imaging system achieves the diffraction limit when the Strehl ratio exceeds 0.8 [12]. At this point, the wavefront aberration of the system should be below λ/14 (rms), and the surface error of the primary mirror should be below λ/40 (rms). However, the rectangular mirror is prone to deformation in complex space environments due to its large aspect ratio, and its surface error may exceed 1 μm (rms), hindering the imaging in the visible light band. If the wavefront information can be detected, the surface shape of the primary mirror can be corrected through active optical technology, improving the image quality.

Shear interferometer, wavefront curvature sensor, pyramid sensor, Shack-Hartmann wavefront sensor, and image-based wavefront sensing are common techniques for measuring wavefront aberrations. Image-based wavefront sensing can estimate the wavefront aberration at the exit pupil of an imaging system from the images captured by a detector without adding extra equipment or introducing non-common path errors. This makes it ideal for space telescopes that demand lightweight and reliable designs. Gerchberg and Saxton [13] proposed the GS algorithm in 1972, which iteratively computes the phase from an image. Based on this, Gonsalves introduced the phase diversity algorithm [14], which recovers the distorted phase information using an objective function that incorporates images from different defocus planes. Since then, the PD method has undergone comprehensive research by institutions such as the University of Central Florida [15], Lockheed Martin Space Systems [16,17], and the Chinese Academy of Sciences [18,19,20], affirming its practicality. This method has already found application in adjusting ground-based telescopes and co-phase detection for segmented mirror telescopes. In this study, we propose extending the application of the PD method to rectangular aperture imaging systems and investigating the effects arising from the noise, aperture aspect ratio, and aberration size.

This paper is organized as follows. Section 2 presents the theory of the PD method. Section 3 introduces the Legendre polynomial and proposes its use for wavefront fitting of rectangular pupil imaging systems. The influence of the secondary mirror obscuration on its orthogonality is also analyzed. Section 4 investigates the effects of factors such as noise, pupil aspect ratio, and aberration size through numerical simulation. Section 5 validates the method using a 280 mm diameter telescope. Section 6 makes discussion.

## 2. Theory of the PD Method

The intensity distribution in the collected image i(x,y) can be expressed as [21]:

(1) i(x,y)=o(x,y)∗PSF(x,y)

The o(x,y) is the distribution function of the observation target. The PSF represents the point spread function of the system in the imaging plane. The ∗ denotes convolution operation. For large-aperture telescopes, the imaging field of view is small. Therefore, the PSF can be assumed to be constant across the entire image plane.

If there is noise n(x,y):

(2) $$i^{*}\left(x,y\right)=i\left(x,y\right)+n\left(x,y\right)$$

When examined in the frequency domain, Equation (2) can be described as:

(3) $$I^{*}\left(u,v\right)=O\left(u,v\right)\cdot OTF\left(u,v\right)+N\left(x,y\right)$$

The $I^{*}\left(u,v\right)$, O(u,v), OTF(u,v) and N(x,y) are the Fourier transform of the i(x,y), o(x,y), PSF(x,y) and n(x,y). The PSF is related to the shape of the pupil and the phase ϕ(x,y) of the wavefront. The ϕ(x,y) contains aberration information. It can be fitted into a two-dimensional polynomial:

(4) $$\phi \left(x,y\right)=\sum_{i=1}^{n}a_{i}\cdot L_{i}\left(x,y\right)$$

where $L_{i}$ is a set of two-dimensional orthogonal polynomial, and $a_{i}$ is the coefficients.

The image intensity distribution does not uniquely determine the system’s wavefront aberration since multiple wavefront phase distributions can produce the same PSF. Therefore, a single image cannot reveal the wavefront information of the imaging system. However, this can be overcome using two images with a known phase difference (typically the in-focus and defocus images), which allows the wavefront information of the system to be retrieved. The relationship between the phases of in-focus image ϕ(x,y) and defocus image $\phi_{d}\left(x,y\right)$ is:

(5) $$\phi_{d}\left(x,y\right)=\phi \left(x,y\right)+△\phi \left(x,y\right)$$

If the phase difference △ϕ(x,y) can be described by the polynomial $L_{i}$, the ϕ(x,y) will be obtained through mathematical methods.

According to the maximum likelihood theory, an evaluation function E(a) is defined to measure the similarity between the reconstructed image and the actual captured image:

(6) $$E\left(a\right)=\sum_{(u,v)\in A}\frac{|I^{*}\left(u,v\right)OTF_{d}\left(u,v\right)-I_{d}^{*}{\left(u,v\right)OTF\left(u,v\right)|}^{2}}{{\left|OTF\left(u,v\right)\right|}^{2}+{\left|OTF_{d}\left(u,v\right)\right|}^{2}}$$

where $I_{d}^{*}\left(u,v\right)$ is the defocus image intensity distribution, and the $OTF_{d}\left(u,v\right)$ is the OTF of the defocus image.

**Figure 1:**
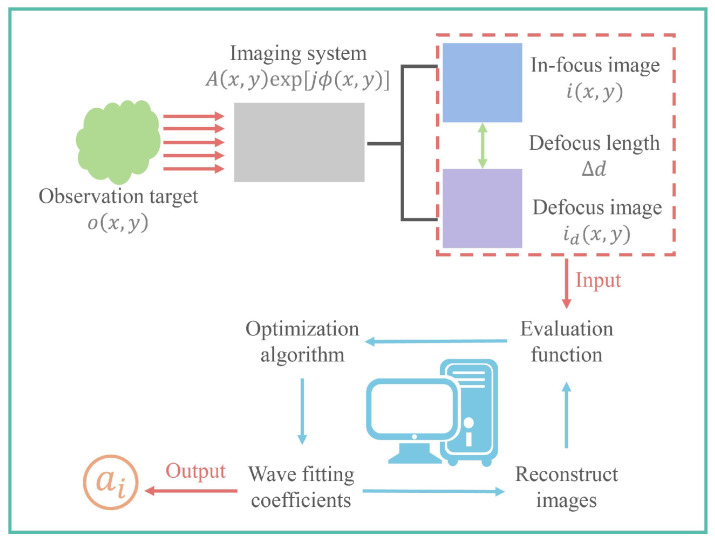
Flow chart of the PD method.

By employing optimization algorithms like genetic algorithms, a set of coefficients $a_{i}$ can be found to minimize the value of the evaluation function. $a_{i}$ can express the phase ϕ(x,y). Figure 1 shows the flow chart of the PD method.

## 3. Wave Fitting Based on the Legendre Polynomial

Zernike polynomials exhibit orthogonality within the circular domain and are frequently employed to model wavefront data in circular optical pupil imaging systems. However, when dealing with a rectangular aperture characterized by substantial length and width, the disparity in shapes between the inner/outer circle and the rectangle results in the loss of orthogonality for Zernike polynomials. In such cases, it is advisable to opt for square-domain orthogonal polynomials for fitting purposes.

Rectangular orthogonal polynomials, such as Legendre and Chebyshev polynomials, can be used for wavefront fitting. And both have defocus terms that can describe the artificial defocus amount accurately.

For a rectangular aperture with length *a* and width *b*, its normalized coordinates are m=x/a and n=y/b, respectively. Table 1 lists the 0th–6th terms of the one-dimensional Legendre polynomial [22].

The orthogonal characteristic of a one-dimensional Legendre polynomial can be formulated as:

(7) $$\int_{-1}^{1}P_{i}\left(m,n\right)P_{j}\left(m,n\right)dm=2\delta_{i,j}$$

The two-dimensional Legendre polynomial $L_{i}\left(m,n\right)$ is obtained by multiplying the one-dimensional Legendre polynomial $P_{xi}\left(m\right)$ and $P_{yj}\left(n\right)$ in the *x* and *y* directions:

(8) $$L_{i,j}\left(m,n\right)=P_{xi}\left(m\right)\cdot P_{yj}\left(n\right)$$

The square domain exhibits the orthogonal characteristic of the two-dimensional Legendre polynomial:

(9) $$\int_{-1}^{1}\int_{-1}^{1}L_{i}\left(m,n\right)L_{j}\left(m,n\right)dmdn=4\delta_{i,j}$$

**Figure 2:**
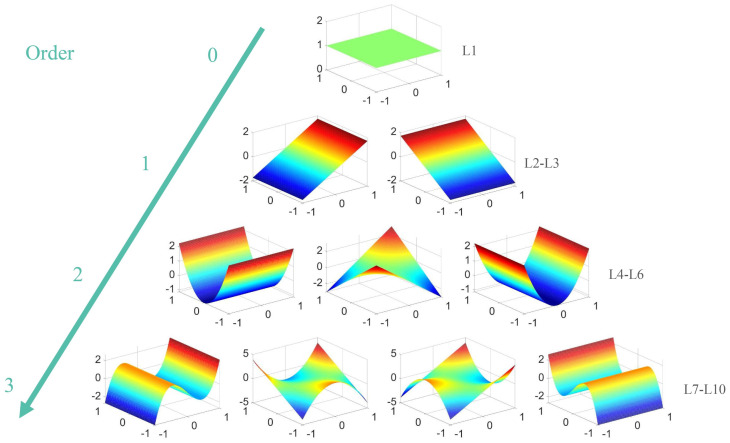
1st–10th terms of the two-dimensional Legendre polynomial. The colors in the graph indicate the values of the polynomial function at each point. Red means positive and blue means negative, and the brighter the color, the larger the absolute value.

Table 2 lists the 1st–10th terms of the two-dimensional Legendre polynomial, and Figure 2 plots the 1st–10th terms.

Table 3 lists the 0–6 order terms of the unnormalized one-dimensional Chebyshev polynomial [23]. It can be seen that the Chebyshev polynomial has a similar form to the Legendre polynomial, except for the coefficients. This implies that the Legendre polynomial and the Chebyshev polynomial have the same order for the same number of terms, and their wavefront fitting ability should be comparable. However, the orthogonality of the Chebyshev polynomials is more complex, and they can be written as Equation (10) on the interval [−1,1]:

(10) $$\int_{-1}^{1}\frac{C_{i}\left(m\right)C_{j}\left(m\right)}{\sqrt{1-x^{2}}}dm=\left\{\begin{array}{c} \frac{1}{2}\pi \delta_{i,j}\;i\neq 0\;or\;j\neq 0\\ \pi \;i=j=0 \end{array}\right.$$

where $C_{i}\left(m\right)$ denotes the *i*–th term of the one-dimensional Chebyshev polynomial. The orthogonality of Chebyshev polynomials requires a weight function $\sqrt{1-x^{2}}$ that depends on x, whereas the weight function of Legendre polynomials is constant. This suggests that the Legendre polynomial is more convenient for fitting the wavefront information when the aspect ratio of the rectangular pupil varies frequently.

## 4. Numerical Simulation

### 4.1. Simulation Imaging

The imaging system has an F-number of 10, a 20 m aperture, a CCD size of 4.4 μm, and operates in the 645.32 nm wavelength range. Specifically, to explore the impact of the aspect ratio of the rectangular pupil, simulation imaging was conducted under varying aperture aspect ratios (*P* = 1, 2, 4, and 8).

**Figure 3:**
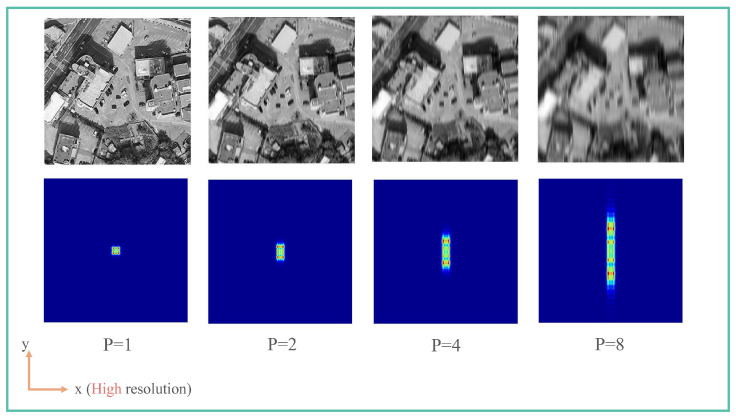
Simulation images and their PSFs with different aspect ratio. PSF values increase with red intensity.

The PSF indicates the ability of an imaging system to focus the energy of a point light source, and its shape reflects the image degradation. For a circular aperture imaging system with high image quality, the PSF is typically a bright spot with high energy concentration. For a rectangular pupil imaging system with a high aspect ratio, the PSF has strong directionality, leading to different resolutions of the image in different directions. Figure 3 displays the simulated images and their PSFs for different aspect ratios. The extended dimension of the aperture was fixed along the x-axis, while the shorter dimension changed along the y-axis according to the aspect ratio. As the aspect ratio increased, the PSF’s span along the y-axis expanded, and the resolution along the y-axis diminished gradually. To preserve enough features, the aspect ratio should be regulated.

The primary mirror’s surface deformation is mainly caused by low-frequency error [24]. Usually, only the first three orders of aberrations are taken into account. They can be fully expressed by the first 10 terms of the Legendre polynomial. Hence, the wavefront aberration was generated by the 4th–10th terms of the Legendre polynomial (excluding piston and tilt, which do not affect the mirror surface shape). The aberration size ranged from 0.5λ to 2.0λ.

Noise affects the images collected by the system. The signal-to-noise ratio (SNR) is often used to measure the impact of the noise. It is defined as [25]:

(11) $$\mathrm{SNR}=10\mathrm{lg}\frac{\sum_{i,j}g{\left(i,j\right)}^{2}}{\sum_{i,j}n{\left(i,j\right)}^{2}}$$

where g(i,j) and n(i,j) represent the gray values at (i,j) of the original image and the noise.

For a top-tier space telescope, the SNR in its captured image typically exceeds 20 dB. In cases where thermal noise is the primary source of interference, it can be characterized by a Gaussian distribution [26] with a mean of 0 and a variance of $\sigma_{n}^{2}$. To enhance realism, a series of remote sensing images with a pixel count of 256×256 were utilized as the target objects, with Gaussian noise introduced to achieve SNR levels of 20 dB (0.01), 25 dB (0.003) and 30 dB (0.001).

The degree of defocus holds significant importance as it directly influences the details in the defocused image, consequently impacting the outcome of the PD method [27]. When the defocus amount is too minimal, the phase difference between the two images becomes negligible, posing challenges in accurately discerning wavefront information. Conversely, excessive defocus results in a substantial loss of high-frequency details in the image, sacrificing valuable features crucial for a successful solution. Optimal results in the PD method are achieved when a change in the defocus amount causes the coefficient of the defocus term corresponding to the long side (the fourth term of the Legendre polynomial) to reach 1.0. Additionally, the coefficient change of the defocus term corresponding to the short side (the sixth term of the Legendre polynomial) is determined as $1/P^{2}$.

### 4.2. The Influence of the Secondary Mirror Obscuration

Coaxial reflective optical structures are common in large-aperture space telescopes, where the secondary mirror obscures part of the primary mirror. This may affect the orthogonality of the Legendre polynomial and lead to a decrease in the detection accuracy of the PD method.

**Figure 4:**
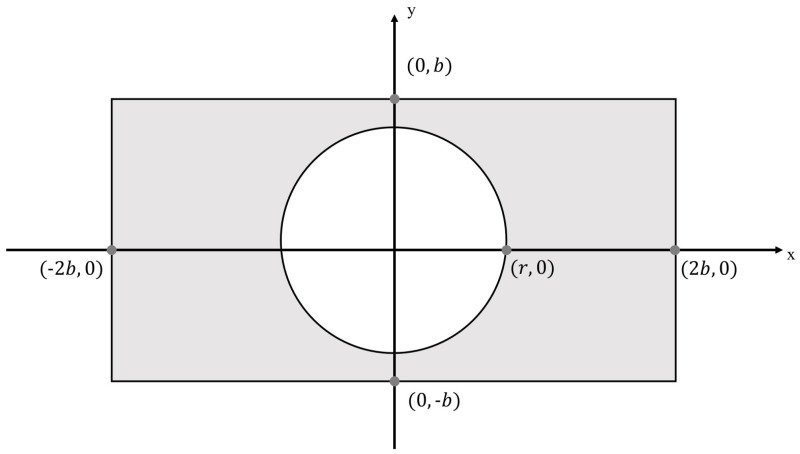
Rectangular region with a circular obscuration.

For the factors of machining technology, the shape of a secondary mirror is usually circular. Assuming a rectangular pupil with an aspect ratio of 2 and a circle obscuration (Figure 4) with a radius of *r* (0<r<b), simulate the aberration of 1.0λ (rms) using the 4th to 10th terms of the two-dimensional Legendre polynomial, add noise with an SNR of 30 dB, and use the PD method to reconstruct the wavefront information.

**Figure 5:**
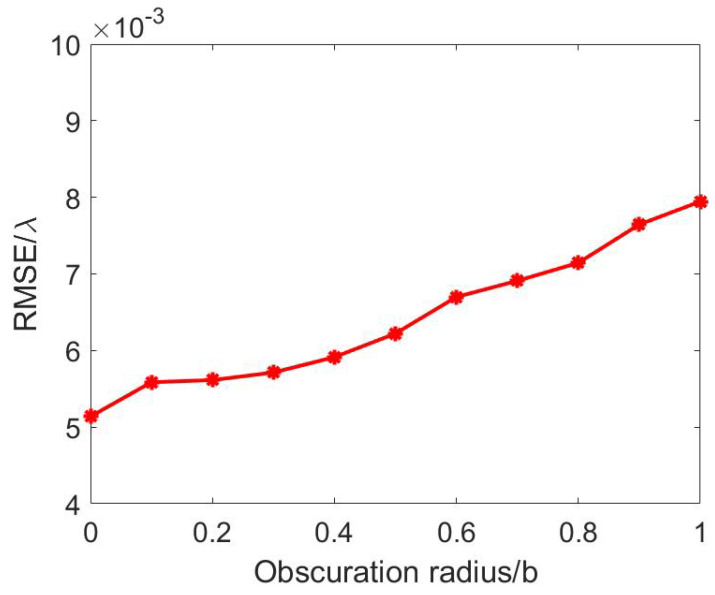
The impact of obscuration size on the calculation accuracy of PD method.

Figure 5 shows the RMSE of the PD method results for different sizes of the obscuration. It can be observed that the PD method becomes less accurate as the size of the secondary mirror increases, but the error always stays below λ/100. Hence, the secondary mirror obstruction is not a significant factor when calculating the wavefront information of the rectangular pupil imaging system using the PD method, especially for large aspect ratios. Therefore, the effect of the sub-mirror obstruction is neglected in the following simulation.

### 4.3. Calculation Results and Analysis

In this paper, aberrated wavefronts with root mean square (RMS) values of 0.5λ, 1.0λ, 1.5λ, and 2.0λ were simulated using the fourth to tenth terms of the two-dimensional Legendre polynomials. Corresponding sharp and blurred images were generated for optical pupil aspect ratios *P* of 1, 2, 4, and 8, respectively. Gaussian noise, based on signal-to-noise ratios of 20 dB, 25 dB, and 30 dB, was incorporated. The wavefront information of the noise was determined using a genetic optimization algorithm, with the algorithm initiating with 350 individuals. To minimize the randomness of the genetic algorithm, the computation concluded when the score of the best individual remained unchanged for 70 consecutive generations.

The calculated wavefront phase is $\phi_{\mathrm{PD}}\left(x,y\right)$. The Root Mean Square Error (RMSE) between the real wavefront $\phi_{0}\left(x,y\right)$ and $\phi_{\mathrm{PD}}\left(x,y\right)$ was used to evaluate the quality of the results. The expression of RMSE is:

(12) $$\mathrm{RMSE}=\sqrt{\frac{1}{N}\sum_{\left(x,y\right)}\left[\phi_{\mathrm{PD}}\left(x,y\right)-\phi_{0}\left(x,y\right)\right]}$$

*N* is the number of sampling points of $\phi_{0}\left(x,y\right)$.

**Figure 6:**
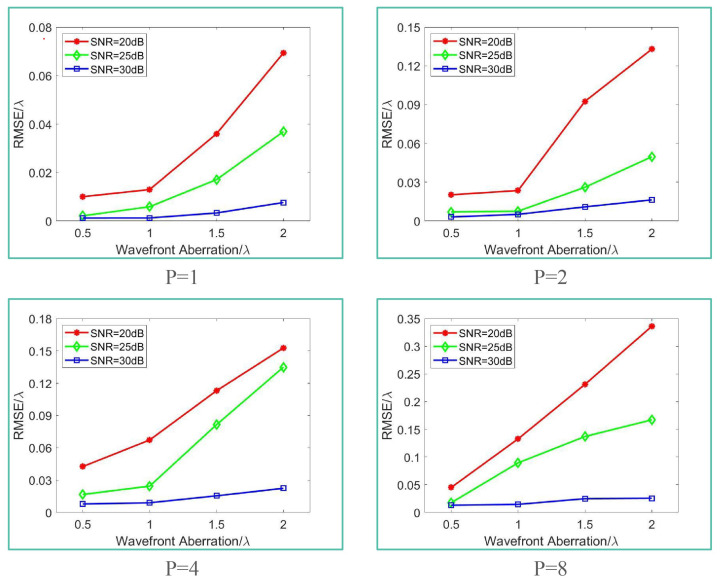
The impact of SNR and aberration size on the calculation accuracy of PD method when *P* = 1, 2, 4 and 8.

Figure 6 shows the RMSE of the PD method calculation results under different conditions. Each data point represents the average outcome of 20 computations. Since the evaluation function is based on the intensity distribution of the image, the spatial features of the image directly affect the calculation accuracy of the PD method. As the pupil aspect ratio, noise, and aberration increase, the image quality decreases, which leads to the decrease in the detection accuracy of the PD method.

When P=1, the PD method achieves the best results. When SNR = 30 dB, even if the aberration is 2λ, the calculation accuracy can reach λ/100, and the relative error (the ratio of RMSE to aberration size) is 0.38%. The calculation accuracy drops slightly when SNR = 25 dB but still reaches λ/100 (relative error 0.59%) when the aberration is less than 1λ. When the aberration reaches 2λ, the calculation accuracy is λ/20 (relative error 2.3%). When SNR = 20 dB, the calculation accuracy worsens, but it remains in the same order of magnitude as when SNR = 25 dB. This indicates that the PD method has good solution results for rectangular pupil imaging systems with a small aspect ratio (similar to circular) when SNR > 25 dB.

When P=2, the accuracy of the PD method deteriorates. The calculation accuracy cannot reach λ/50 when SNR = 20 dB and aberration is greater than 0.5λ. It is necessary to use active optical devices to correct the aberration or improve the SNR to enhance the calculation accuracy and meet the wavefront detection requirements.

When P=4, the accuracy of the PD method declines further, and this effect is more pronounced when P=8. The detection accuracy is only at the sub-wavelength level when the aberration is greater than 1λ. However, it is noteworthy that when P=8, SNR = 25 dB, and aberration size is 0.5λ, the calculation accuracy reaches within λ/50. This indicates that for imaging systems with large pupil aspect ratio and good SNR, although very accurate detection of the wavefront is impossible when the aberration is large, it is feasible to use active optical devices to perform multiple rounds of detection-correction operations on the system wavefront and ultimately achieve the image quality standard.

## 5. Experiment

**Figure 7:**
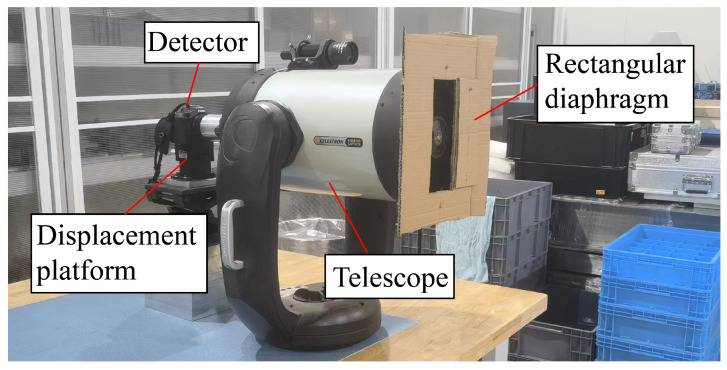
Imaging device. A Celestron CGEM 1100HD telescope became a rectangular imaging system with a rectangular diaphragm. A Canon R50 camera was connected to the displacement platform as the detector.

The experimental setup employed a Celestron CGEM 1100HD telescope (made by Celestron, a company located in Torrance, CA, USA) with a 280 mm aperture and an F-number of 10. A rectangular diaphragm featuring an aspect ratio of 2 was added to create a rectangular aperture. Multiple imaging systems with rectangular pupils can be obtained by rotating the diaphragm. Additionally, a filter with a center wavelength of 645.32 nm was incorporated into the telescope, and a Canon R50 camera (made by Canon, a company located in Tokyo, Japan.) was connected to the displacement platform to collect in-focus and defocus images. Refer to Figure 7 for a visual representation of the telescope.

The device captured numerous images with varying focus levels. A broader region featuring the identical observation target was captured and aligned, and then a 256×256 image was extracted based on the alignment outcomes. This extracted image was subsequently employed in the PD method for wavefront calculation. The degree of defocus was established at 0.5163 mm in accordance with Equation (13).

(13) $$\varepsilon_{z}=-8F^{2}△d$$

**Figure 8:**
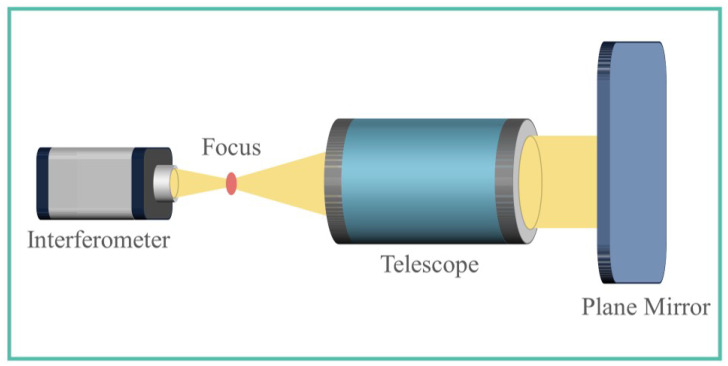
Schematic diagram of self-collimating examination.

The self-collimating examination accurately identifies the wavefront, serving as a benchmark for assessing the efficacy of the PD method. Figure 8 illustrates the underlying principle and the practical implementation of the self-collimating test. In this setup, an interferometer releases a spherical wave from the imaging system’s focal plane. This wave transforms into a plane wave after traversing the imaging system and is then reflected by a plane mirror. Retrieval of the wavefront information occurs when the light wave returns to the interferometer through its initial optical pathway.

**Figure 9:**
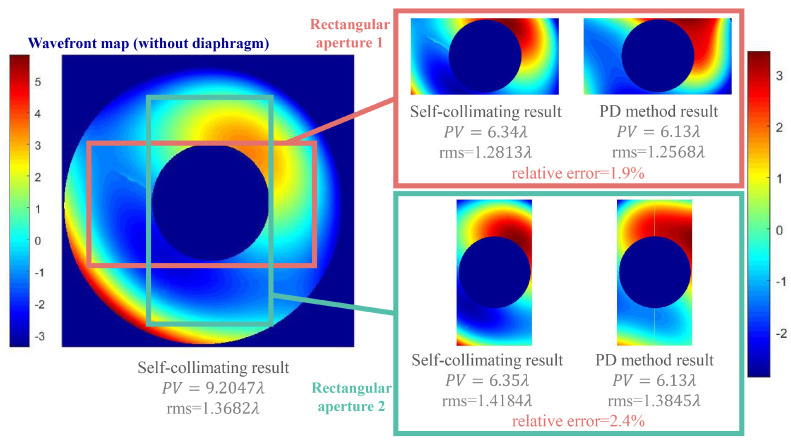
Comparison of wavefront map gained by the two methods.

By rotating the diaphragm, multiple imaging systems with rectangular pupils were obtained. Figure 9 displays the wavefront maps identified through both the PD method and the self-collimating test in two sets of experiments. In the two groups of experiments shown in the figure, the PD method detected the wavefront deviation from the self-collimating detection result as 1.9% and 2.4%. The detection accuracy was slightly lower than the simulation results (about 1.7%) due to the neglect of higher-order aberration, defocus error, secondary mirror obstruction, and other factors. This indicates that in the rectangular pupil imaging system (especially the system with active optical devices), using the PD method to detect the wavefront information and then correct the surface shape of the primary mirror is feasible.

## 6. Discussion

In summary, this paper explored how to apply the PD method to the imaging system with a rectangular aperture. For wavefront fitting, we propose the Legendre polynomial, which is orthogonal in the rectangular region and has defocus terms (the fourth and sixth terms) that make it more suitable for the PD method than other polynomials. When ignoring the higher-order aberration, the first 10 terms of the Legendre polynomial can satisfy the requirements for wave fitting.

The PD method retrieves the wavefront information from both in-focus and defocus images and reconstructs it by an evaluation function and an optimization algorithm. We simulated the detection accuracy under different aspect ratios, aberration sizes, and SNRs to explore the application range. These factors significantly affect the intensity distribution of the images, reduce the spatial feature information, and lower the wavefront detection accuracy. The results were satisfactory when the SNR was above 25 dB. This implies that we should enhance the robustness of the PD method and the quality of the images. The pupil aspect ratio and the aberration also affected the results. We should control the pupil aspect ratio to improve the detection accuracy. Large initial aberration is a major challenge, but it can be corrected by active optics technology through several detection-correction cycles.

The large secondary mirror in the experiment reduces the Legendre polynomial orthogonality, lowering the PD method accuracy. Moreover, to keep the aperture area, the rectangular aperture aspect ratio is at most 2. We will design and produce a better, less obscured, and higher aspect ratio rectangular aperture imaging system and explore its wavefront detection further.

This paper demonstrated the applicability of the PD method for wavefront detection in the imaging system with a rectangular pupil. To use this method in space telescopes, further research on the mechanisms of various errors and precise defocus mechanisms is required.

The rotation of the primary mirror in the RSA system will cause a motion blur on the PSF, and the wavefront detection technology for this process needs to be explored in the future. Moreover, during the practical imaging procedure, the rotation of the RSA pupil around the center introduces known rotation-related phase differences in the captured images, offering an additional avenue for utilizing the PD method in calculating wavefront information. 

## Figures and Tables

**Table 1:** The 0th–6th terms of the one-dimensional Legendre polynomial.

Term	Expression	Aberration
0	1	Piston
1	$\sqrt{3}m$	Tilt
2	$\left(\sqrt{5}/2\right)\left(3m^{2}-1\right)$	Defocus
3	$\left(\sqrt{7}/2\right)\left(5m^{3}-3m\right)$	
4	$\left(3/8\right)\left(35m^{4}-30m^{2}+3\right)$	
5	$\left(\sqrt{11}/8\right)\left(63m^{5}-70m^{3}+15m\right)$	
6	$\left(\sqrt{13}/16\right)\left(231m^{6}-315m^{4}+105m^{2}-5\right)$	

**Table 2:** The 1st–10th terms of the two-dimensional Legendre polynomial.

Term	Expression	Aberration
1	1	Piston
2	$P_{X1}P_{Y0}$	X-tilt
3	$P_{X0}P_{Y1}$	Y-tilt
4	$P_{X2}P_{Y0}$	X-defocus
5	$P_{X1}P_{Y1}$	
6	$P_{X0}P_{Y2}$	Y-defocus
7	$P_{X3}P_{Y0}$	
8	$P_{X2}P_{Y1}$	
9	$P_{X1}P_{Y2}$	
10	$P_{X0}P_{Y3}$	

**Table 3:** The 0th–6th terms of the unnormalized one-dimensional Chebyshev polynomial.

Term	Expression	Aberration
0	1	Piston
1	*m*	Tilt
2	$2m^{2}-1$	Defocus
3	$4m^{3}-3m$	
4	$8m^{4}-8m^{2}+1$	
5	$16m^{5}-20m^{3}+5m$	
6	$32m^{6}-48m^{4}+18m^{2}-1$	

## Data Availability

The data presented in this study are available on request from the corresponding author.
